# Continuous Monitoring of Shelf Lives of Materials by Application of Data Loggers with Implemented Kinetic Parameters

**DOI:** 10.3390/molecules24122217

**Published:** 2019-06-13

**Authors:** Bertrand Roduit, Charles Albert Luyet, Marco Hartmann, Patrick Folly, Alexandre Sarbach, Alain Dejeaifve, Rowan Dobson, Nicolas Schroeter, Olivier Vorlet, Michal Dabros, Richard Baltensperger

**Affiliations:** 1 AKTS SA, Technopôle 1, 3960 Sierre, Switzerland; c.luyet@akts.com (C.A.L.); m.hartmann@akts.com (M.H.); 2 armasuisse, Science and Technology Centre, 3602 Thun, Switzerland; Patrick.Folly@ar.admin.ch (P.F.); alexandre.sarbach@ar.admin.ch (A.S.); 3 PB Clermont EURENCO Group, Rue de Clermont, 176-4480 Engis, Belgium; a.dejeaifve@eurenco.com (A.D.); r.dobson@eurenco.com (R.D.); 4 School of Engineering and Architecture of Fribourg, HES-SO University of Applied Sciences and Arts Western Switzerland, Bd de Pérolles 80, 1700 Fribourg, Switzerland; nicolas.schroeter@hefr.ch (N.S.); Olivier.Vorlet@hefr.ch (O.V.); Michal.Dabros@hefr.ch (M.D.); Richard.Baltensperger@hefr.ch (R.B.)

**Keywords:** shelf life, internet of things, IoT, data loggers, advanced kinetic analysis, vaccines, propellants, mean kinetic temperature

## Abstract

The evaluation of the shelf life of, for example, food, pharmaceutical materials, polymers, and energetic materials at room or daily climate fluctuation temperatures requires kinetic analysis in temperature ranges which are as similar as possible to those at which the products will be stored or transported in. A comparison of the results of the evaluation of the shelf life of a propellant and a vaccine calculated by advanced kinetics and simplified 0th and 1st order kinetic models is presented. The obtained simulations show that the application of simplified kinetics or the commonly used mean kinetic temperature approach may result in an imprecise estimation of the shelf life. The implementation of the kinetic parameters obtained from advanced kinetic analyses into programmable data loggers allows the continuous online evaluation and display on a smartphone of the current extent of the deterioration of materials. The proposed approach is universal and can be used for any goods, any methods of shelf life determination, and any type of data loggers. Presented in this study, the continuous evaluation of the shelf life of perishable goods based on the Internet of Things (IoT) paradigm helps in the optimal storage/shipment and results in a significant decrease of waste.

## 1. Introduction

One of the most important daily-life uses of kinetic investigations of the thermal behavior of materials is the possibility of the application of the computed kinetic parameters for the prediction of materials’ properties at temperatures higher or lower than those used during data collection. Everybody is faced a few times each day with labels indicating the shelf life of items. Buying any daily-use products, storing chemicals, propellants, medicines, and thousands of temperature-sensitive products, one checks or carefully monitors the date of their validity expressed by common expressions, such as “best before” or “expiry date”. The information depicting the period of time in which the item’s properties fulfill certain criteria is of great importance, not only for individuals, but also has great economic significance. The improper handling of a batch of, e.g., expensive vaccines may result in the waste of millions of dollars or may be dangerous for the population. According to the World Health Organization, the losses associated with temperature excursions in health care come to ca. 35·10^9^ USD per year [1]. It is therefore obvious that enormous efforts are undertaken in order to monitor the conditions of the handling (storage) of any kind of product and to evaluate the impact of time–temperature parameters on the material properties.

The problem of the deterioration of the products’ properties is extremely complicated, due to the fact that many parameters influence the rate of their deterioration. However, in general, independently of the kind of material, one of the main parameters influencing the shelf life is the temperature, and general suggestions given by producers inform users about the temperature range and duration of storage. More and more often, especially in large-scale handling, the monitoring of the temperature, as a decisive factor of the material’s stability, is conducted by electronic devices (data loggers) that continuously collect the temperature–time data, which allow for the evaluation of the correctness of the storage.

The estimation of the aging extent belongs to a typical problem of the application of kinetic analyses in solving daily-life problems. The evaluation of the kinetic parameters of the main deterioration process allows the prediction of the rate of this process under any temperature fluctuations. However, in many cases, such a kinetic analysis is much more difficult to perform than during common kinetic measurements carried out in laboratories when the experimental time is limited to a few hours and when thousands of experimental data may be collected. The kinetic description of the process based on experimental points collected in the temperature (or time) domain, which is significantly different than that in which the properties of investigated material are of interest, may not be precise enough. Therefore, an additional difficulty in the application of the kinetic approach for the prediction of the shelf life of products arises from the fact that the kinetic parameters should be evaluated in the temperature ranges which are as similar as possible to those at which the products will be stored (or transported) in. This, in turn, results in very time- and effort-consuming experiments to supply the number of data points necessary, which is generally a few orders of magnitude smaller than the data collected in common kinetic experiments. These sparse data have to be elaborated by specific kinetic and statistic approaches in order to give the “best model combinations” with meaningful prediction bands which could be successfully applied during a shelf life [2]. In any case, such a kinetic analysis has to be validated by some experimental data lying in the considered time–temperature domain in which the product is stored.

In the present study, we propose the merging of the time–temperature profiles with the modified kinetic approach described in our previous paper [2], which is well suited for the estimation of the kinetics of the deterioration of any temperature-sensitive products. Our proposal is illustrated by the determination of the shelf lives of products which are relatively temperature resistant, such as propellants, and those which are very sensitive to temperature excursions, such as vaccines. These two classes of compounds can be used as examples representing the boundary conditions occurring during storage and the procedures applied for the evaluation of their aging kinetics can be used for almost all kinds of products.

The knowledge of temperature and its fluctuations is a very important factor for the estimation of the shelf life of materials, therefore, its continuous monitoring during storage or shipping has become more common. Information collected by data loggers, among other methods, monitor storage (shipping) temperatures to ensure their quality by checking whether the temperature of sensitive products is in accordance with approved temperature specifications.

The correct estimation of the material’s shelf life can be performed only in the case when both the correct kinetic parameters and temperature data are known. Therefore, it is of great importance to combine the temperature–time data, which are collected and stored in the data loggers, with a kinetic analysis of the deterioration process. The application of the data loggers to the monitoring of only the temperature may be significantly extended this way, by a much more precise evaluation of the deterioration extent of products.

This study will address the advantages of implementing kinetic parameters into data loggers, allowing continuous online monitoring of the change of material properties. Additionally, it will be illustrated that simplified applications of the Arrhenius equation, such as in the form of the mean kinetic temperature approach or predictions based on the 0th or 1st order kinetic models, may result, in certain cases, in imprecise results.

## 2. Results and Discussion

### 2.1. Experimental

Stability tests of materials based on different experimental techniques monitoring their properties are performed by artificial aging either at (i) one single temperature or (ii) at a number of different temperatures (multi-temperature aging procedure). The single-temperature aging procedure is cheaper, however, as it is generally based on simplified assumptions, although it cannot precisely assess the safe storage life at an arbitrarily chosen temperature. The multi-temperature aging [3,4] procedure is more time- and effort-consuming, however, it enables a more precise prediction of a material’s behavior for a wide range of temperature profiles after the calculation of the kinetic parameters and the selection of the best kinetic model, which can be verified by using, for example, the Akaike and Bayesian information criteria [2,5,6]. It may give the answer if a material is sufficiently stable to be stored for a given period of time, for example, 10 years at a specific temperature, such as ambient storage conditions, e.g., 25 °C (t_25_ ≥ 10 years), or for more specific temperature and time profiles. Based on the bootstrap sets of estimated kinetic parameters, the prediction can be enhanced by the estimation of the prediction band (PB) in the form of, e.g., the upper and lower 95 percentiles (PB 95% confidence). The simulations presented in this study were done with the AKTS-Thermokinetics Software [5].

### 2.2. Kinetic Analysis

#### 2.2.1. Determination of the Reaction Rate and Kinetic Triplets

The reaction progress can be defined as follows:

(1) $$\alpha =\frac{Y-Y_{0}}{Y_{end}-Y_{0}},$$

where *Y*, *Y_0_*, and *Y_end_* represent the value characterizing the certain material property at time *t*, *t* = 0, and *t* = *t_end_*, respectively.

The residual sum of squares (RSS) can be used to compute the parameters used for simulation:

(2) $$RSS={\sum_{i=1}^{N}\left(Y_{i,\exp}-Y_{i,cal}\right)}^{2},$$

where the indices represent the value of an experimental point and its calculated value and *N* is the total number of points collected discontinuously. The conversion rate is expressed as:

(3) $$\frac{d\alpha }{dt}=A\cdot \exp\left(-\frac{E}{R}\cdot \frac{1}{T}\right)f(\alpha ),$$

where *t* is the time, *T* is the temperature, *R* is the gas constant, *E* is the activation energy, *A* is the pre-exponential factor, α is the reaction extent, and f(α) is a differential form of the conversion function depending on the reaction model.

Although there is a significant number of various reaction models, f(α), they all can be reduced to three major types when considering the dependence of the reaction progress on the time in isothermal conditions: Accelerating, decelerating, and S-shaped (logistic or sigmoidal function). Each of these types has a characteristic “reaction profile” or “kinetic curve”, the terms frequently used to describe a dependence of α or dα/d*t* on *t* for a given *T*.

In the present study, we applied the S-shaped model:

(4) $$f\left(\alpha \right)={\left(1-\alpha \right)}^{n}\alpha^{m}.$$

Under isothermal conditions, such sigmoidal reaction models may be considered as accelerating at the beginning (when α is close to 0) and decelerating at the reaction end (when α is close to 1) so that the process rate reaches its maximum at some intermediate values of the extent of the conversion. The sigmoidal reaction model turns into the nth order model (decelerating type under isothermal conditions) for *m* = 0; and to the 0th or 1st order models if *m* = 0 and *n* = 0 or 1, respectively. This means that the quality of fit of multi-temperature aging data by the sigmoidal reaction model is always equally good (if *m* = 0) or better (if *m* ≠ 0) than those obtained with the nth order model. Sigmoidal reaction models may also be used to describe accelerating type reactions if *n* = 0 and *m* > 0. One should note that the solution of the reaction rate (Equation (4)) using the ’sigmoidal reaction model’ if both *n* and *m* ≠ 0 implies the presence of a very small amount of reaction progress, α_0_, at time *t* = 0. In this study, we assumed that α_0_ amounts to 1 × 10^−10^.

Despite the applied model in kinetic analysis, the number of data points should be larger than the number of fitted parameters applied. The application of too many parameters which are fitted to a small number of data points leads to overfitting, which is manifested by the nonsensical values of the calculated parameters and the reduced predictive performance of the model. Methods based on information theory, such as the Akaike and Bayesian information criteria (AIC and BIC) [2,6,7] and, recently, [8], can be used to assess the statistical relevance of the fitted parameters, *n* and/or *m*, and find the optimum number of parameters.

#### 2.2.2. Propellants: Application of Kinetic Analyses for Shelf Life Predictions

Using nitrocellulose-based propellants as an example, it is necessary to consider that the decomposition products may influence their chemical stability. In order to prevent these undesired processes, small amounts of stabilizing compounds are added to the propellants in order to react with the decomposition products, therefore preventing their reactions with the parent material. Surface modification of the propellant during aging may also change the ballistic properties and the shelf life of propellants. The basic information concerning the assessment of the stability of propellants and safe lifetime are presented in the study of de Klerk [9].

The optimal procedure for the investigation of a propellant’s aging should be based on the application of more than one experimental technique monitoring changes of the material during storage. The main reason for analyzing different properties is the fact that certain material properties are inherently less stable than others and can vary differently during temperature excursions.

The determination of the kinetics of the investigated phenomena is difficult due to the fact that the number of experimental points in rationally limited periods of time is relatively small. To perform a meaningful kinetic analysis, having few experimental points collected at only two or three temperatures, we propose the significant optimization of the experimental procedure required for the correct kinetic description of the investigated process. The optimization of the experimental procedure is based on decreasing temperature–time domains which, in turn, allows avoidance of the necessity of collecting experimental points during a few months or years. Using the proposed method, it is possible to verify the selection of the best kinetic model and computed kinetic parameters by the experimental points collected after several days or even years by checking if they are lying inside the prediction bands. After successful validation of the kinetic analysis with experimental data, it was possible to uncover the differences of the reaction course for the various propellant properties in different climates and storage.

Aging can give rise to many phenomena, which may modify the thermal behavior of composite propellants. The aim of our study was to compare the results obtained during the investigation of the artificial aging performed by different analytical techniques in which different physico-chemical phenomena occur in the material. We applied our kinetic and statistical approach to the results obtained by four different methods in which specific material behaviors were monitored, namely: The pressure firing (PF), gas evolution (VST), stabilizer depletion (UPLC) [10], and the heat evolution (HFC) [11].

**Figure 1:**
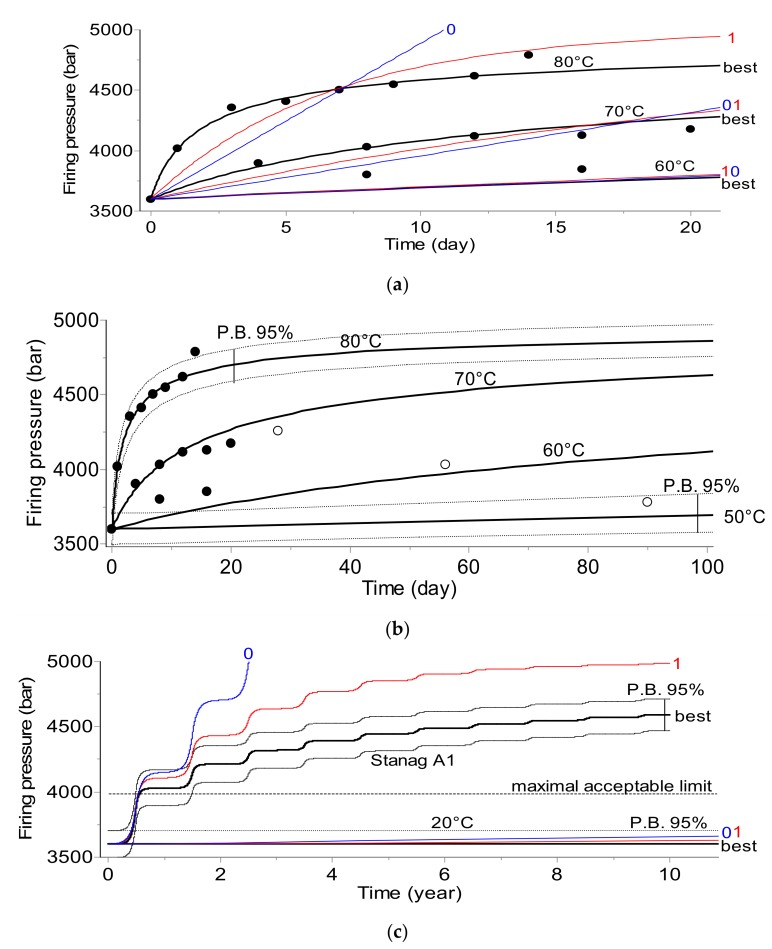
Pressure firing (PF) test. (**a**) Predictions of pressure firing peak values based on 15 experimental points (solid symbols) collected during 20 days in the temperature range of 60–80 °C. Predictions based on the best model are marked in bold, numbers 0 and 1 placed on the solid lines depict the predictions based on the 0th and 1st reaction order, respectively. (**b**) Prediction curves at 50, 60, and 70 °C were verified by the experimental points marked by the open circles. The prediction bands (dotted lines) were determined by the bootstrap method. (**c**) Comparison of the prediction using the best, 0th, and 1st order models at 20 °C over 10 years and for climatic category A1 (diurnal seasonal storage) according to STANAG 2895. The arbitrarily chosen acceptable limit of each measured quantity is marked by a dashed line.

**Figure 2:**
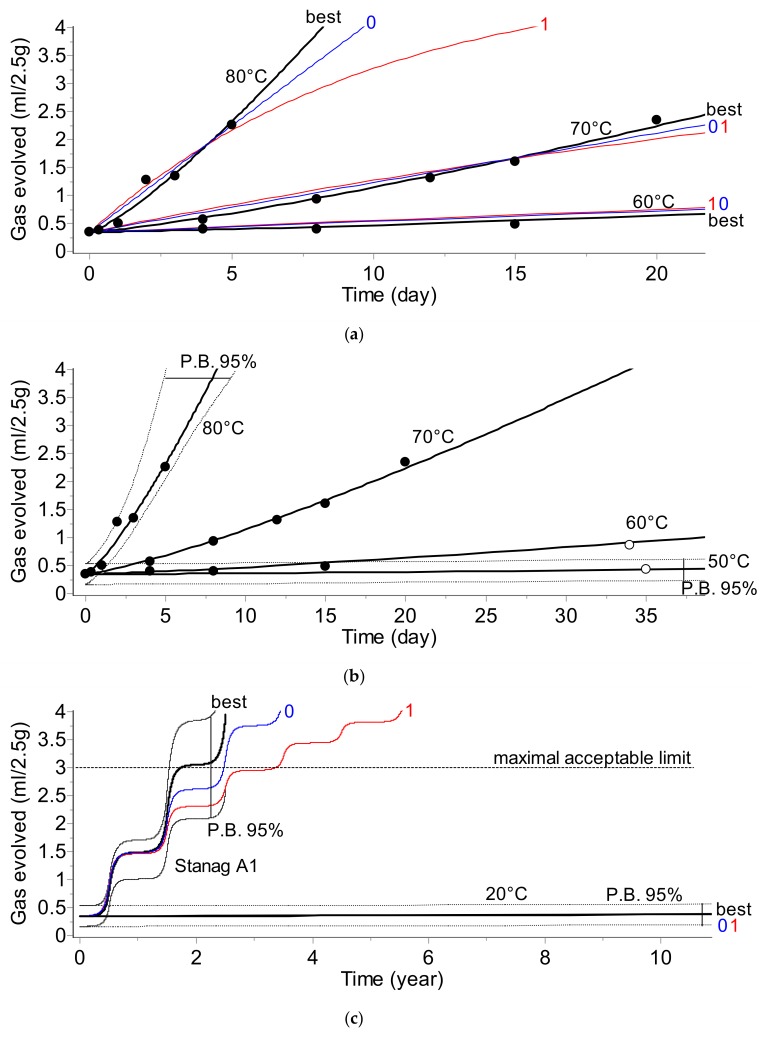
Gas evolution (VST) test. The plots present: (**a**) The fit of experimental data by the best (bold), 0th, and 1st order models. (**b**) Prediction of the long-term reaction course for temperatures of 80, 70, 60, and 50 °C (predictions are verified by experimental data marked as empty circles). (**c**) 10-year predictions for 20 °C and climate category STANAG A1.

**Figure 3:**
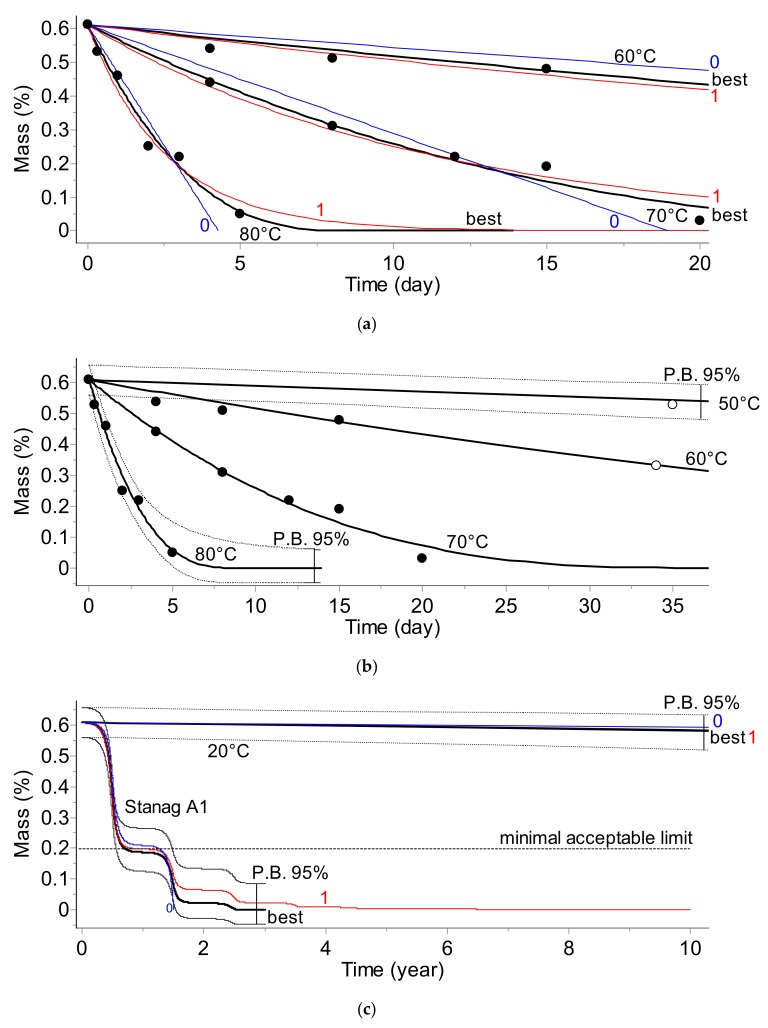
Stabilizer depletion (UPLC) test. (**a**) The fit of experimental data by the best (bold), 0th, and 1st order models. (**b**) Prediction of the long-term reaction course for temperatures of 80, 70, 60, and 50 °C (predictions are verified by experimental data marked as empty circles). (**c**) 10-year predictions for 20 °C and climate category STANAG A1.

**Figure 4:**
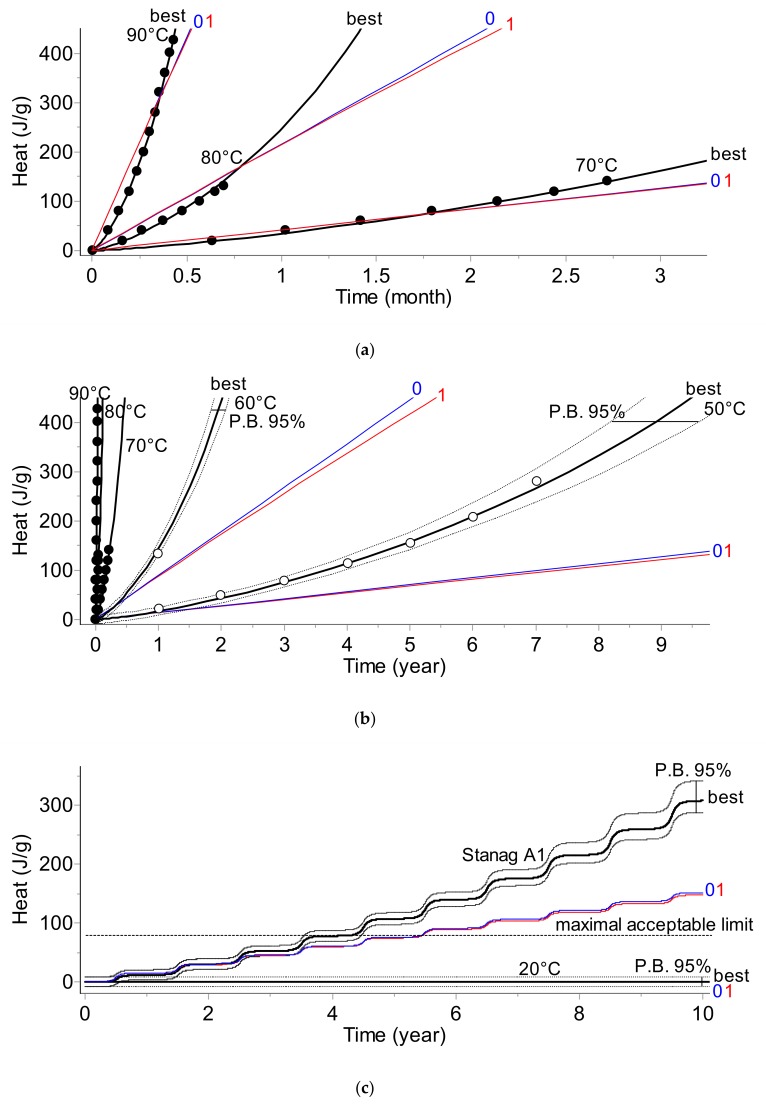
Heat evolution (HFC) test. (**a**) The fit of experimental data collected at 90, 80, and 70 °C by the best (bold), 0th, and 1st order models. (**b**) Prediction of the long-term reaction course for temperatures in the range of 90–50 °C. The predictions for 60 and 50 °C are verified by experimental data marked as empty circles collected once a year. (**c**) 10-year predictions for 20 °C and climate category STANAG A1.

The results of the kinetic analysis for all applied testing methods are depicted below in Figure 1, Figure 2, Figure 3 and Figure 4. The plots display:

- Top section: Fit of experimental data at three temperatures (solid circles) by the best model chosen according to Akaike (AIC) [2,6] and Bayesian (BIC) [2,7] criteria and by commonly applied 0th and 1st order kinetic models (curves are marked as «best», «0», and «1», respectively).
- Middle section: Long-term prediction of the reaction course according to the best model containing prediction bands with 95% confidence. The empty circles indicate the results of the additional experiments not used during the kinetic analysis which were applied for the verification of the simulations. The plot additionally contains the simulated course of the reaction at a lower temperature (50 °C) with one experimental point.
- Bottom section: Comparison of the prediction of the reaction course at 20 °C over 10 years and for climatic category A1 (diurnal seasonal storage according to [10] using the best, 0th, and 1st order models.

The results of the kinetic analysis based on AIC and BIC criteria are displayed in Table 1 for four analytical methods: PF, VST, UPLC, and HFC, respectively.

Results depicted in Figure 1, Figure 2, Figure 3 and Figure 4 clearly indicate that the application of the simplified assumptions concerning the form of the kinetic function, f(α), may influence the predictions of the shelf life of the propellants. In the two analytical methods, namely gas evolution monitoring and stabilizer depletion, this difference is not large; however, the pressure firing and, especially the heat evolution test recommended by STANAG 4582, the predictions based on simplified kinetics (assuming 0th or 1st order reaction) differ significantly for the results obtained by advanced kinetic analysis. For example, for the climate category, STANAG A1, the shelf life evaluated for the advanced kinetics amounts to circa 4 years whereas the predictions based on first order kinetics result in a shelf life of ca. 5.2 years (Figure 4c). Depending on the applied method chosen for monitoring the propellant properties (PF, VST, UPLC, or HFC), the *E* values were in the range of 138.6 to 206.5 kJ·mol^−1^.

The presented results clearly show that the kinetic parameters which are going to be implemented into the data loggers should be evaluated by an advanced kinetic analysis. The combination of the time–temperature data with simplified kinetics may result in an imprecise evaluation of the aging extent.

#### 2.2.3. Pharmaceuticals

##### The Peculiarities of the Application of Kinetics for the Evaluation of the Shelf Life

Evaluation of the thermal stability of pharmaceuticals is a difficult task due to the influence of numerous factors on the rate of their deterioration. These factors include the thermal stability of the active component; interaction between active ingredients and excipients; methods of packing; and temperature, light, and moisture conditions encountered during storage, shipment, and handling. Pharmaceuticals should at all times be stored under the conditions recommended by the manufacturer to prevent deterioration which can result in a loss of potency and efficacy. Certain medications, such as vaccines and biological medicines, need to be stored under refrigeration in order to maintain the stated potency and ensure safety of the product until its expiry date.

Over the past several decades, numerous scientific papers and books have addressed the problem of the evaluation of the shelf life of pharmaceuticals, see, for example, the book edited by Carstensen and Rhodes [12], comprehensive reviews of Kartoglu and Milstien [13], the book of Waterman [14] and his papers [15,16], or recent publications of Fan et al. [17], Fu et al. [18], Almalik et al. [19], Faya et al. [20], Khan et al. [21], Clénet et al. [22,23], or Clancy et al. [24].

The determination of the shelf life of medicines is an important task of the pharma-industry and the World Health Organization (WHO), whose primary role is to direct international health within the United Nation’s system [25,26]. It is also important for institutions attempting to supply medicines to populations in countries where transport and storage facilities do not fulfill the criteria required for the preservation of products requiring cold chain management. The term “cold chain” refers to the transportation and storage of drug products, such as vaccines, insulin, and biological medicines requiring stable refrigerated conditions. The proteins present in these products often have vulnerable structures and their unfolding at higher temperatures significantly decreases the medicine’s activity.

An evaluation of the change in the medicine’s potency during storage and transportation is generally based on recording their temperature which should follow the approved profiles. The list of WHO-recommended temperature-monitoring devices for storage and transportation [27] contains electronic shipping indicators, vaccine vial monitors, and user-programmable temperature data loggers.

The “controlled temperature chain” (CTC) is an innovative approach to vaccine management, allowing vaccines to be kept at temperatures outside of the traditional cold chain of +2 to +8 °C for a limited period of time under monitored and controlled conditions, as appropriate to the stability of the antigen. A CTC typically involves a single excursion of the vaccine into ambient temperatures not exceeding +40 °C for a period of time not shorter than 3 days [28].

In order to be sure that vaccines have not been exposed to temperatures higher than +40 °C, a “peak threshold indicator” must accompany the vaccines at all times when, in a CTC, the temperature exposure of the vaccines is monitored. This indicator is a card with a sticker, which changes color from light grey to black as soon as the temperature exposure has exceeded +40 °C. If this is the case, all vaccines in that vaccine carrier must be discarded, following an appropriate investigation and documentation of the event. Additionally, temperature monitoring’s impact on the vaccines’ potency retention is carried out by means of vaccine vial monitors (VVMs). A VVM is a label containing a heat-sensitive material which is placed on a vaccine vial to register cumulative heat exposure over time. The combined effects of time and temperature cause the inner square of the VVM to darken, gradually and irreversibly. A direct relationship exists between the rate of color change and temperature. Vaccine vial monitors and peak temperature threshold indicators protect potency and quality by monitoring cumulative and peak exposure to heat.

##### Shelf Life Evaluation Criteria Derived from the Arrhenius Equation

In a more advanced procedure of monitoring the thermal behavior of vaccines, the mean kinetic temperature (MKT) is used. This method was introduced by Haynes et al. [29] who addressed the fact that climate-based temperature variation in uncontrolled pharmaceutical storage makes it difficult to select a single temperature for use in product expiry testing. A detailed description of the MKT concept can be found in [30], its modification in [31], and limitations in [32], where one finds the recommended caution in using the MKT to evaluate temperature excursions.

The MKT is defined as a single calculated temperature at which the total amount of degradation over a particular period is equal to the sum of the individual degradations that would occur at various temperatures. The MKT may be considered as an isothermal storage temperature that simulates the non-isothermal effects of storage temperature variation, i.e., that corresponds to the same kinetic effects of a time–temperature distribution. The calculation gives increased weighting to higher temperature excursions than normal arithmetic methods, recognizing the accelerated rate of thermal degradation of materials at higher temperatures. The commonly used formula for MKT calculation was introduced by Haynes and is given by:

(5) $$T_{MKT}=\frac{\Delta H/R}{-\ln\left(\frac{e^{\frac{-\Delta H}{RT_{1}}}+e^{\frac{-\Delta H}{RT_{2}}}+\ldots +e^{\frac{-\Delta H}{RT_{n}}}}{n}\right)},$$

where T_MKT_ is the mean kinetic temperature in degrees Kelvin, Δ*H* is the activation energy in kJ·mol^−1^, R is the gas constant in J·mol^−1^·K^−1^, T_1_ to T_n_ are the temperatures at each of the sample points in degrees Kelvin.

Continuous evaluation of the MKT seems to better characterize the impact of time–temperature parameters on the rate of deterioration of products rather than, for example, the mean temperature recorded during long-term storage (shipment). However, the application of the concept of the MKT has significant drawbacks from the point of view of advanced kinetics. Restrictions, such as the necessity of collecting data in the same time-intervals, the assumption that the activation energy amounts to 83.144 kJ·mol^−1^, or that the reaction of the deterioration is a one-step first order reaction, indicate that the MKT approach is roughly linked with advanced kinetic analysis. A default value of 83.144 kJ·mol^−1^ is typically used because it is supposed to be an acceptable approximation for most pharmaceutical compounds. According to Seevers et al. [30], it is an average value of activation energy for breaking most covalent bonds. However, according to [32], for a wide range of pharmaceuticals, *ΔH* in Equation (5) may be in the range of 42 to 125 kJ·mol^−1^. Furthermore, the application of the 1st order model as a rule may result in an incorrect evaluation of the aging extent for most products (see, e.g., Figure 4).

The information received from data loggers, vaccine vial monitors, peak temperature threshold indicators, and other devices recommended by the WHO [27] are the only indicators as to whether the storage fulfills a specific refrigeration temperature criterion. Therefore, the implementation of continuous evaluation of the MKT into data loggers does not change the situation in which it is impossible to continuously monitor the actual degree of the deterioration of samples. The MKT is essentially just another way to express the impact of temperature during sample exposure which does not bring information of whether the permissible reaction progress limit is reached. This issue can be solved only if the kinetics of the deterioration or inactivation process can be merged with the time/temperature profiles recorded by data loggers. Application of the MKT concept for more complicated temperature profiles is described in the papers of Okeke at al. [33,34].

Although a comprehensive discussion of the problem of the kinetics of medicine inactivation is beyond the scope of this study, numerous scientific papers have appeared to better address this issue, see, e.g., Kumru at al. [35] or WHO guidelines [25]. However, general remarks concerning the specific treatment of sparse data collected during stability studies of medicines should be addressed because an evaluation of the kinetic parameters of medicines’ deterioration differs from commonly applied kinetic workflows (see [2,22,23] and the references cited herein). One of the main reasons for this situation is the fact that an evaluation of the kinetics of the degradation of vaccines can be done only in time- and effort-consuming experiments and therefore the number of data points which could be applied in kinetic analysis is relatively small, often in the range of 20 to 30. This, in turn, requires modification of the kinetic and statistical approaches applied during standard kinetic analysis [2].

We are aware that a variety of methods of vaccine stability testing (such as biological assays or chemical and physical studies) may be applied for evaluating vaccine immunogenicity or efficacy changes. We treat this issue from the kinetics point of view by considering the change of the chosen parameter which is used for the evaluation of vaccine potency or activity after its normalization in the range of 0 to 1 (or 0–100%), which is commonly applied in typical kinetic studies. Kinetic analysis applied for the evaluation of vaccine degradation rates is generally carried out by the accelerated degradation test in which the investigated products are exposed to temperatures greater than those recommended for vaccine storage (typically 5, 25, or 37 °C). During the kinetic analysis of the data, often, the simplified kinetics models, such as 0th or 1st order kinetic functions, are applied. Such models fail to correctly describe the complicated course of decomposition of biological materials, which frequently show complex and multi-step degradation behavior (see [23] and the references cited herein). The rate constant derived from simplified models is often of little value during an advanced kinetic workflow; the prediction of half-lives of vaccines only from the Arrhenius plot depicting the rate of material degradation [36] is not precise enough because it is based on only one of three required kinetic parameters, namely the activation energy. The two other equally important kinetic parameters, i.e., the pre-exponential factor in the Arrhenius equation, *A*, and the form of the kinetic function, f(α), are not considered in simplified kinetic approaches. A more precise kinetic description of the decomposition of biological compounds [37,38] was obtained with kinetic parameters of two-step models, which better mimic the complicated decomposition of the investigated samples. The application of the autocatalytic kinetic model in the evaluation of the shelf life of pharmaceuticals was presented in the review by Brown and Glass [39].

For illustration purposes, we used the pharmaceutical product studied by an advanced kinetic approach for which the kinetic parameters are known. A freeze-dried measles vaccine was investigated in our former study [2] for which the criteria for the discrimination of the best kinetic models were done using the AKTS-Thermokinetics Software [5] and based on the information theory introduced by Akaike [6] and its Bayesian counterpart [7]. The deterioration rate of this pharmaceutical product, called throughout our study the “model vaccine”, is characterized by the following kinetic parameters (see Table 5 in [2]) used in all our simulations:

(6) $$\frac{d\alpha }{dt}=6.95\cdot 10^{19}\cdot exp\left(-\frac{156.26\cdot 10^{3}}{RT}\right){\left(1-\alpha \right)}^{2}+2.28\cdot 10^{12}\cdot exp\left(-\frac{121.13\cdot 10^{3}}{RT}\right).$$

**Figure 5:**
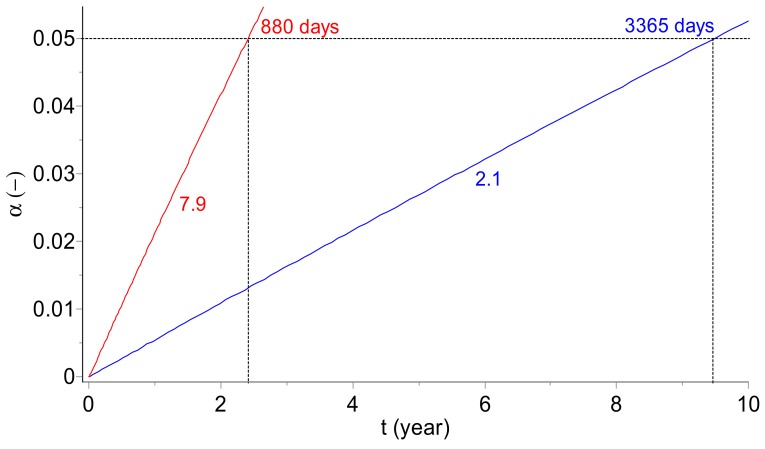
Model vaccine: Simulation of the long-term prediction of the change of the viral titer degree at temperatures of 2.1 and 7.9 °C (marked on the curves) lying in the range of 2 to 8 °C applied during the cold chain storage. Note the severe change of the vaccine shelf life (set arbitrarily as a time when 5% of the viral titer loss is reached) from 880 to 3365 days for 7.9 and 2.1 °C, respectively, despite fulfilling the cold chain criterion in both cases.

The kinetic simulations for the course of deterioration of the model vaccine kept in the temperatures of 2.1 and 7.9 °C, i.e., those lying in the commonly applied temperature range characteristic for the “cold chain”, are displayed in Figure 5. The presented results show that the expression “cold chain” is very imprecise from a kinetics point of view. For the boundary temperatures characteristic of the cold chain (2 °C < T < 8 °C), the shelf lives of a model vaccine may significantly vary from 880 to 3365 days.

**Figure 6:**
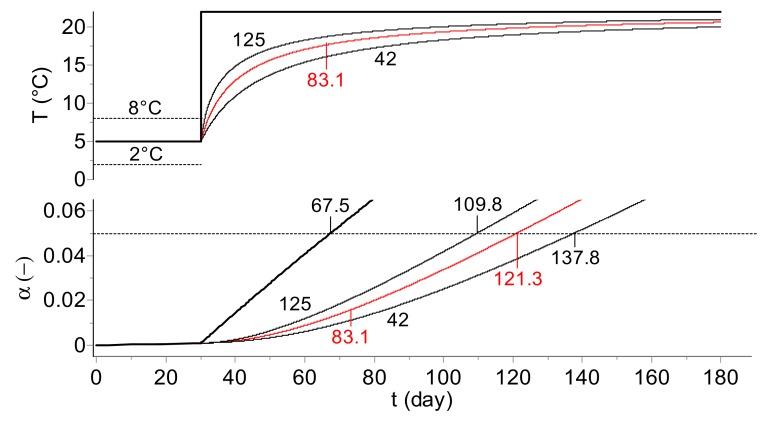
Model vaccine: Simulation of the long-term prediction of the change of virus infectivity (infectious titer) during the temperature excursion from 5 to 22 °C (TOR procedure). Top plot: time dependence of the real (bold) and MKT temperatures, bottom plot: the degree of infectious titer for the real (bold) and MKT temperatures. The mean kinetic temperatures were calculated for three activation energy values (marked on the curves in kJ·mol^−1^). Note that application of the MKT leads to the overestimation of the shelf life: the value of 5% of degradation is reached after 67.5 days if following the real temperature, whereas according to the MKT, depending on the assumed activation energy, this loss of infectious titer is reached after ca. 110, 121, and 138 days for *E* = 42, 83.1, and 125 kJ·mol^−1^, respectively.

**Figure 7:**
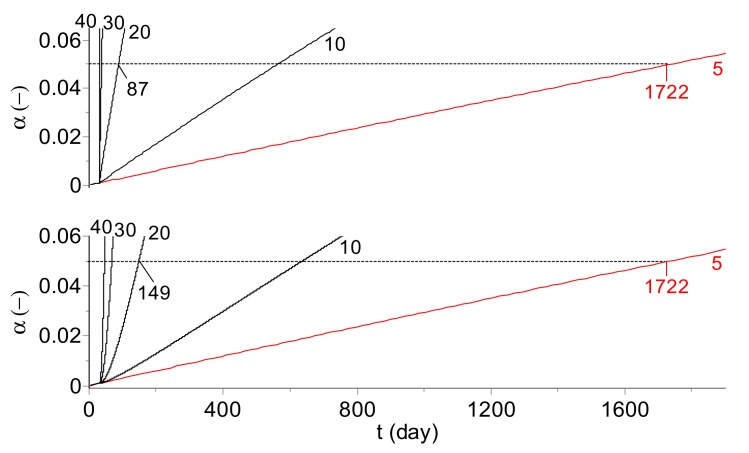
Model vaccine: The change (α) of virus infectivity during 30 days at 5 °C followed by the temperature excursions (10, 20, 30, and 40 °C marked in °C on the curves), for the real temperature course (top) and for MKT temperatures calculated by the assumption that *E* = 83.144 kJ·mol^−1^ (bottom). Bold curves show the vaccine deterioration progress at 5 °C, the dependences temperature vs. time for the real temperature and the MKT are identical at a constant temperature. The predicted limit of the shelf life, set arbitrarily as a time when a 5% loss of the infectious titer is reached, amounts at 5 °C to ca. 1722 days.

To illustrate the possible pitfalls resulting from the application of a simplified MKT approach, we present the simulations of the deterioration of samples of the “model vaccine” kept at 5 °C for 30 days followed by a rapid temperature excursion to 22 °C (Figure 6) and to 10, 20, 30, and 40 °C (Figure 7). A comparison of the real reaction progress with those based on the temperatures obtained by the computed MKT indicates the severe differences in time to reach the same extent of vaccine decomposition (see Figure 6). For a viral titer, the read-out is generally expressed in Log(pfu/vial). To be in line with the commonly applied expression of the extent of the material deterioration in the kinetic studies, instead of the viral titer in Log(pfu/vial), the reaction progress, α, is used in this study together with an arbitrarily chosen critical value of 5% for the permissible limit of degradation following a temperature excursion out of, e.g., the “cold chain”. The application of the real kinetics for time out of refrigeration (TOR), which is very important in pharma logistics, enables precise quantification of the impact of a temperature excursion on the reaction progress, which amounts to 5% (arbitrarily set limit for the shelf life considerations) after 67.5 days. However, the predicted times to reach the same level of decomposition are considerably overestimated and amount to ca. 110, 121, and 138 days when including into Equation (6) the MKT for *E* = 42, 83.1, and 125 kJ·mol^−1^, respectively.

Figure 7 displays the extent of the infectious titer of the model vaccine occurring during 30 days of storage in the cold chain at 5 °C, followed by the temperature excursions to 10, 20, 30, and 40 °C. The top part of the plot depicts the reaction extent (calculated according to Equation 6) for the real temperature whereas in the bottom plot the MKT (calculated for *E* = 83.144 kJ·mol^−1^) has been used for the reaction course estimation. For a temperature excursion of 20 °C, as presented in Figure 7, the shelf life amounts to 87 or 149 days at the real temperature and MKT, respectively. The results shown in Figure 6 and Figure 7 indicate that not only the arbitrarily assumed value of the activation energy used for MKT calculation but also the difference between the real temperature and evaluated MKT influence the determination of the correct shelf life value. 

### 2.3. Continuous Shelf-Life Estimation by Using Data Logger

**Figure 8:**
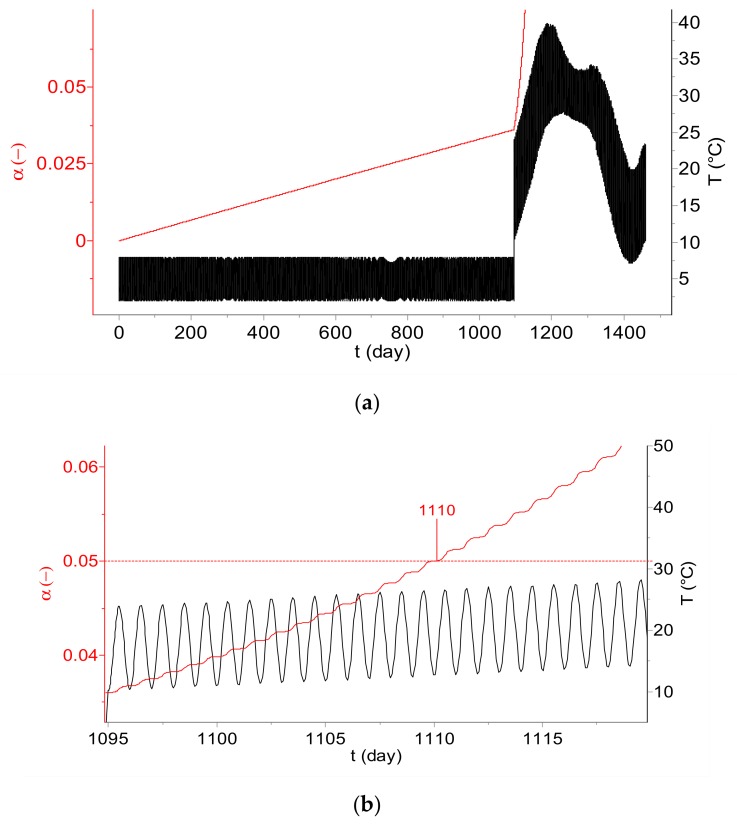
(**a**) Degree of the loss of infectious titer, α, after 3 years of storage in the cold chain (temperature variations between 2 and 8 °C), followed by storage at a real atmospheric temperature profile corresponding to the month of March in New Delhi (India). (**b**) The storage period between 1095 and 1120 days. The shelf life is reached 15 days after removal from the cold chain.

The AKTS-Thermokinetics Software [5] allows the determination of the reaction extent, α (in our case the degree of the infectious titer change), in any temperature mode. Figure 8 shows the dependence of α on the time during temperature variations during a 3-year long storage in a cold chain (2 °C < T < 8 °C), followed by storage at ambient conditions with daily temperature fluctuations corresponding to the month of March in New Delhi (India). After three years of storage in the cold chain, the reaction extent amounts to ca. 3.6%. After removal from the cold chain and exposure of the vaccine to ambient temperature fluctuations, the shelf life (=5%) is reached after 15 days (see Figure 8b).

**Figure 9:**
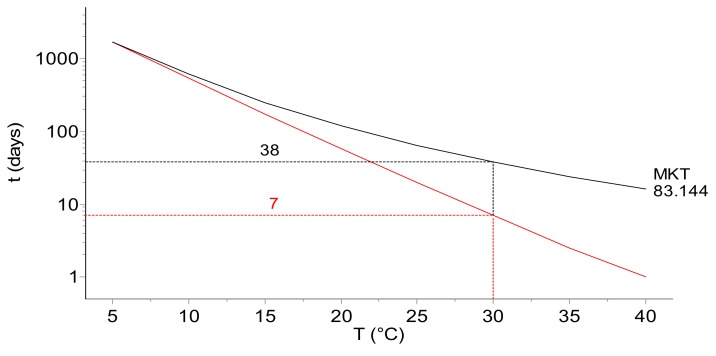
TTT (transformation–time–temperature) diagram presenting the shelf life as a function of the value of the excursion temperature after 30 days of storage in the cold chain (5 °C). The simulations show the dependences for the real temperature (bold) and the MKT calculated by the assumption of the activation energy as *E* = 83.144 kJ·mol^−^^1^. At the excursion temperature of 30 °C, the infectious titer loss, set arbitrarily to 5%, is reached after ca. 7 days. Note that the application of the MKT leads to severe overestimation of the shelf life. Depending on the assumed values of the activation energy, the same infectivity loss is reached after ca. 51 days for *E* = 42, 38 days for 83.1, and 29 days for 125 kJ·mol^−^^1^, respectively.

Online determination of the reaction extent (degree of the infectious titer change) based on the kinetic parameters and temperature–time data is of great importance because it allows immediate evaluation of the remaining shelf life as a function of the excursion temperature. This can be done using the TTT (transformation–time–temperature) plot presented in Figure 9, in which the position of the oblique bold line calculated for the 5% reaction extent allows immediate determination of the time at which, for an arbitrarily chosen temperature (30 °C on the plot), the loss of infectious titer reaches the set value of 5%.

Such a TTT diagram allows quick evaluation of how many days at a specific constant temperature (in the depicted case, in a range of 5 to 40 °C) the vaccine removed from the cold chain will fulfil the required criterion of usability. The duration of the shelf life of the model vaccine kept 30 days at 5 °C after exposition to 30 °C, evaluated from the presented plot, amounts to 7 days for the real temperature and to 38 days for the MKT. Also, in this case, the arbitrary choice of the *E* value required for the MKT evaluation changes the predicted value of the shelf life: The virus infectivity loss at 30 °C after one-month storage at 5 °C estimated for the *E* values of 42, 83.1, and 125 kJ·mol^−1^ will amount to 51, 38, and 29 days, respectively. All these values are significantly overestimated when compared to the 7 days predicted for the real temperature course. A similar but simplified concept of the TTT diagram, which was based on the 1st order kinetics only, was presented by Ammann [40].

**Figure 10:**
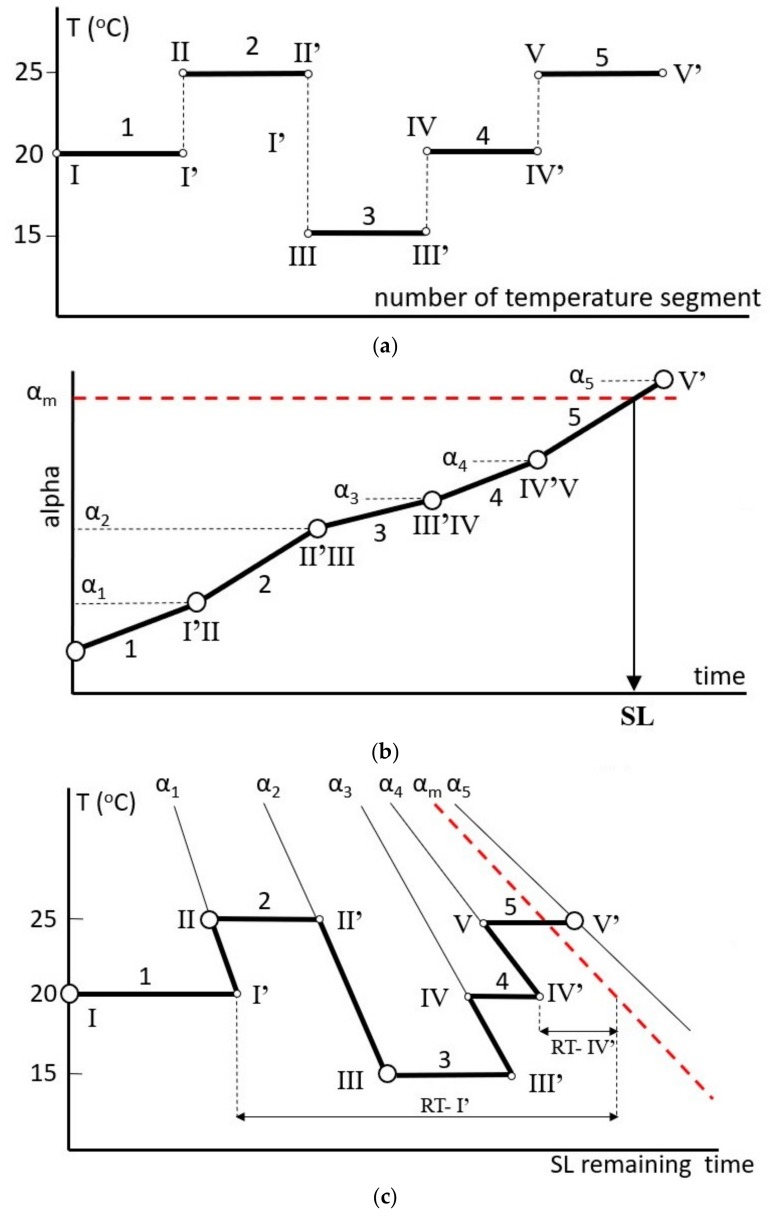
Scheme of the estimation of the remaining shelf life of the model vaccine during temperature variations. (**a**) Time–temperature dependence during the storage; (**b**) change of the reaction extent, α, as a function of the time and temperature profile shown above, the dashed line presents the maximal acceptable degradation extent which determines the shelf life value; (**c**) TTT plot presenting the mutual dependence of the time, temperature, and reaction progress. The dashed line (plot 10b) depicts the maximal acceptable degradation degree and their intersection with the actual degradation extent indicates the time in which the shelf life (SL) is reached. The determination of the remaining shelf life during storage displayed in Figure 10c is explained in the text.

The TTT diagram depicted in Figure 9 can be applied for quick evaluation of the duration of the shelf life only at a constant temperature. The procedure proposed in this study allows for its application under any temperature mode. In our approach, the reaction extent (whatever parameter or approach is used for the evaluation of this parameter) is calculated continuously online at any customized period of time. Knowing at any time the reaction extent and the actual temperature, it is possible to continuously evaluate the remaining time until the sample reaches the shelf life value. The scheme of our approach is presented in Figure 10. For the sake of clarity, the concept is illustrated by isothermal temperature variations in an arbitrarily chosen range of 15 to 25 °C in relatively long periods of time. During real data logger applications, the presented long isothermal steps are replaced by the short time periods set by the user, allowing for the application of the TTT approach at any temperature mode.

Figure 10a presents the set of temperature segments recorded by the data logger. In segment no.1, the temperature of the sample amounts to 20 °C and at its end, the reaction extent (degree of the deterioration) reaches the value of α_1_ (see Figure 10b). The sample aging progress displayed in Figure 10c occurs along the horizontal line at a temperature of 20 °C, which at the end of segment no.1 crosses the isoconversional line for α_1_ at the point I’.

Immediately after the end of segment no.1 begins segment no. 2, which occurs at 25 °C. The change in temperature from 20 to 25 °C results in a shift from I’ to II, which depicts the point at which the sample has the decomposition extent, α _1_ at a temperature of 25 °C. The sample aging in segment 2 proceeds along the horizontal line II–II’ at 25 °C and at the end of segment (point II’), the sample aging progress reaches the value of α_2_. In segment no. 3 on the TTT diagram, point II’ moves to the position marked by III, which presents the intersection of the isoconversional line of α_2_ with the horizontal line at the temperature of 15 °C. The further stages of the sample aging occur according to the presented scenario. The determination of the remaining time (RT) to reach the shelf life value is explained (for the sake of clarity) for segments 1 and 4 only, which proceed at a temperature of 20 °C. The length of the horizontal line drawn at the temperature of the respective stage from point I’ at the end of segment 1 till the intersection with the isoconversional line drawn for the maximal allowed the reaction extent (marked by α_m_) amounts to RT-I’ and at the end of segment 4 (point no. IV’) to RT-IV’. The shelf life is reached before the end of segment no.5.

The simplified example presented in Figure 10 is used only for the basic explanation of the concept of the remaining shelf life calculations. In real applications, these values (characterized by the lengths of the arrows, RT-I’ and RT-IV’) are calculated continuously at the end of each segment collected by the data logger. The frequency of the data collection is arbitrarily set by the user.

**Figure 11:**
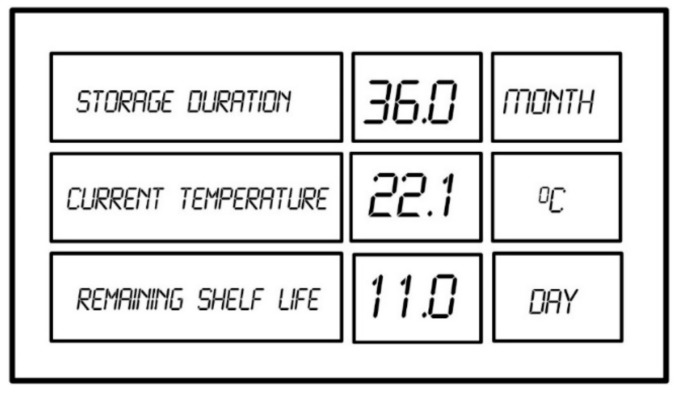
Online information concerning the remaining shelf life received from the data logger.

The typical information displayed from the data logger concerning the online evaluation of the remaining shelf life is shown in Figure 11.

**Figure 12:**
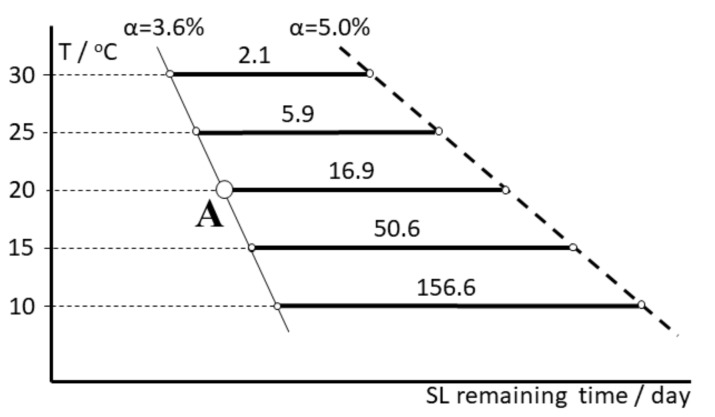
Dependence of the shelf life (SL) remaining time on the isothermal temperature which allows the prediction of the usability of the sample stored at 20 °C with a deterioration progress of 3.6% (point A). The remaining values of the shelf life at temperatures between 10 and 30 °C are displayed in days on the thick horizontal lines.

The implementation into the data logger of the TTT diagram based on the kinetic parameters also allows online prediction of the dependence remaining shelf life–isothermal temperature for the arbitrarily chosen variable. Figure 12 illustrates the basic concept of how the remaining time is evaluated at any temperature for the sample at a temperature of 20 °C and having the deterioration progress of α = 3.6% (point A).

**Figure 13:**
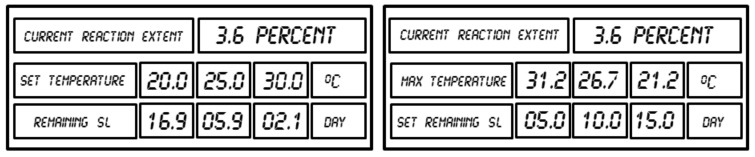
Display of the shelf life remaining for the sample with a deterioration progress of 3.6% at the user set temperature (left) or the maximal isothermal temperature for the shelf life remaining time (right) set by the user.

After increasing the temperature from 20 to 30 °C, the remaining time decreases from 16.9 to 2.1 days, and decreasing the temperature from 20 to 10 °C leads to increasing the shelf life remaining to 156.6 days. It is also possible to arbitrarily set the value of the SL-remaining time (e.g., 5.0 days) and determine the value of the maximal temperature at which this period of time is not exceeded and the vaccine remains within the approved shelf life specifications. The examples of data displayed by the data logger for both scenarios are shown in Figure 13.

In summary, we would like to underline that the chosen methods of estimating the shelf life of pharmaceuticals presented in this study are used for illustration purposes only. The proposed procedure of implementing and merging kinetic analysis with continuously recorded time–temperature data is independent of the chosen criteria of the shelf life definition, storage and/or shipment conditions, applied kinetic approach, procedures used during accelerated stability studies, etc. We are aware that dozens of institutions, organizations, and scientific groups have already published an enormous number of papers, documents, and regulations concerning the above issues. It is not our goal to participate in discussions about the definition of the shelf life, such as those presented in the paper of Capen et al. [41]. Presenting our simulation, we keep in mind that the temperature is only one out of a few parameters influencing the deterioration rate of pharmaceuticals and the real rate of deterioration may depend on the relative humidity or other parameters. However, this fact does not change the general conclusion arising from our study, which is the following: The best available comprehensive kinetic analysis implemented into data loggers together with continuously monitoring of time–temperature data allows for continuous monitoring of the remaining shelf life of medicine products. The application of the kinetics based on a simplified 0th or 1st order kinetic approach may lead to imprecise predictions. Our study illustrates that it is advantageously possible to determine the exact time during which a batch of the product is expected to remain within the approved shelf life specifications for any temperature fluctuations.

### 2.4. Basic Technical Information about the Application of Data Loggers with Implemented Kinetic Data

This study describes the implementation of advanced kinetic parameters into data loggers which collect time–temperature data belonging to the “Internet of Things (IoT)” approach introduced in 1999. The term, IoT, refers to an evolution in computer technology and communication aiming to connect objects together via the Internet through either wired, wireless, or hybrid systems. The IoT enables anytime, anywhere, anything, and any media communications, for example, information can be sensed about the environment, such as temperature, humidity, localization, light exposure, etc. The interaction and communication among “smart things/objects” is reliable and occurs in real-time. A comprehensive review concerning IoT is presented in [42], and its general application is described in [43]. A general application of IoT for monitoring perishable goods is given for cold chain management [44,45] and specific applications in the area of healthcare are shown in [46,47] and in [48,49] for agriculture. Due to the rapidly increasing number of applications of IoT, the McKinsey’s Global Institute predicts that IoT will have an economic impact of between 4 and 11 × 10^12^ USD by 2025 [50].

According to [51], monitoring systems in the current market involve the recording of only raw environmental data, e.g., temperature and/or humidity. Only rarely are these used for controlling environment parameters throughout the storage and transportation process. However, perishable goods (such as, e.g., the vaccines used by us for illustration purposes) are degraded during transportation to customers due to longer delivery routes and improper handling methods or the necessity of removal from the cold chain environment. An enormous amount of data is collected in the monitoring system. There is, however, little attention given towards further investigation of transforming the data to predict the current online quality of goods, such as, e.g., the deterioration extent or the actual shelf life value.

In our study, we presented a solution in which not only the environmental data (we use temperature as an example) but also the on-time degradation extent is monitored. Technical details of the application of data loggers are beyond the scope of this study, therefore only the main information, which can be interesting for non-specialists, were given.

The SensorTag developed by Texas Instruments was applied in this study, however, the described concept is not limited to any specific data logger supplier or this type of electronic device. The data logger reads various experimental conditions at regular time intervals, such as temperature and/or relative humidity. After some adjustments, customization and optimization of the original firmware by AKTS for SL prediction purposes, all readings can be stored in the memory.

Communication with a SensorTag and its programming is done by a smartphone (or similar device) and the AKTS mobile app. All key information, such as the kinetic parameters, the permissible limits of the goods’ properties, the duration of the intervals between two successive readings, etc., are transmitted wirelessly via Bluetooth using a customized application and stored in the data logger. The concept is not restricted to Bluetooth communication.

We applied Bluetooth technology essentially because it has become a standard for (i) wireless transmission of data, (ii) availability and accessibility, (iii) ease of use, and (iv) energy efficiency. Once launched and the data logger starts recording required experimental conditions, it is possible to store ca. 10,000 data in its memory. To save battery, customized firmware switches to sleep mode automatically between two successive readings. It is also possible to put the device in sleep mode for a specific period of time so that it can be transported without restrictions by airplanes. Due to merging time–temperature data with the kinetic parameters implemented in the memory, the extent of aging can be computed and displayed continuously for the thermal history provided by the electronic sensor. Information about the aging extent may be displayed in different modes:

(i) Through small LED lights, whose displays may be set up to vary depending on the reaction progress computed online.

(ii) Option (i) can be enhanced by using a smartphone which, in proximity of the SensorTag and after identification through a QR code, can access wirelessly the stored temperature readings and display graphically the evolution of the temperature profile and determined reaction progress of the investigated property of the goods.

(iii) Tracking information can be sent to a central computer where the AKTS-Datalogger Manager Software continuously collects all readings submitted by smartphones or similar transmitters (after data encryption and QR code identification) along with geo-location information.

All authorized senders and receivers have instant access to collected information, which allows the temperature and deterioration extent of stored (shipped) products to be maintained within approved temperature specifications.

## 3. Materials and Methods

### 3.1. Propellant

As a model sample, the nitrocellulose-based propellant was used. All analyses were carried out in compliance with the NATO operational procedures.

#### 3.1.1. Analytical Methods

##### Pressure Firing (PF)

The PF testing method is based on the measurement of the maximum pressure during firing. In general, the measuring system employs a piezoelectric transducer flush mounted in the chamber of the test barrel. The pressure developed by the gases from the burning propellant exerts a force on the transducer through the cartridge case wall, causing the transducer to deflect, and creating a measurable electric charge which, after calibration, is converted into the pressure. The propellant aging increases the maximum pressure value.

The measurements were performed with materials aged at 60, 70, and 80 °C using a home-made experimental arrangement. The data obtained for propellant aged at 50 °C were used for verification of the simulation procedure. The pressure was measured by a piezo-resistive pressure sensor while speed measurements were done by the photoelectric sensor placed in the light barrier.

##### Gas Evolution (VST)

VST is based on the measurement of the volume of gas evolved on heating under specified conditions. The sample was heated in an evacuated tube and the volume of gas evolved was determined by either the mercury manometric method or by using a pressure transducer.

Experiments were carried out using a tube Vacuum Stabilizer Tester (OZM Research, Hrochův Týnec, Czech Republic) equipped with a Pressure Transmitter DMP 331 (BD Sensors GmbH, Thierstein, Germany). The measurements were performed with materials aged at 60, 70, and 80 °C. The data obtained for propellant aged at 50 °C were used for verification of the simulation procedure.

##### Ultra-Performance Liquid Chromatography (UPLC)

UPLC was used for stability testing by chromatographic monitoring of the stabilizer concentration which in fresh propellant amounts to ca. 1% and decreases during propellant aging. This method allows monitoring of the amount of stabilizer and allows detection if its concentration in the propellant does not drop below a safety level. The testing procedure fulfills the requirements AOP-48 Ed.2 [10]. UPLC analyses were carried out on a UPLC-MS Acquity chromatograph (Waters, Milford, MA, USA) with the following experimental settings: Column Acquity UPLC BEH C18 1.7 μm 2.1 × 50 mm, isocratic mode, column temperature of 45 °C, flow rate of 0.7 mL·min^−^^1^, injection volume of 0.5 μL, and measuring time of 1.2 min. The measurements were performed with materials aged at 60, 70, and 80 °C. The data obtained for propellant aged at 50 °C were used for verification of the simulation procedure.

##### Heat Flow Calorimetry (HFC)

HFC monitors the evolution of heat during the decomposition of the propellants, allowing detection of the heat generation in the μW range. With such a high sensitivity, it is possible to investigate the early stages of decomposition, i.e., for the reaction progresses, α, in the range from 0 to 0.05. Due to the fact that we have the results of very long experiments at a relatively low temperature (50 °C, 7 years), the results obtained by means of HFC were used for the validation of our kinetic and statistical approaches. In the kinetic analysis depicted in this study, the duration of the experimental domain was restricted to 3 months and the maximal heat evolved was lower than 450 J·g^−1^. Kinetic analysis was performed with arbitrarily chosen discontinuous data in the range of 70 to 90 °C. The testing procedure based on HFC is described in STANAG 4582 [11]. The measurements were performed using a TAM IV microcalorimeter (TAInstruments, New Castle, DE, USA). Data of the samples aged at 70, 80, and 90 °C were used for kinetic analysis. Additional experiments carried out once a year at 50 and 60 °C were used for verification of the simulation results.

### 3.2. Vaccine

A freeze-dried live-attenuated virus measles vaccine was used in our study as a model substance.

#### 3.2.1. Stability Monitoring

The kinetics of the deterioration of the vaccine were studied by the plaque assay method by determining the infectious level of virus preparations. The virus titers are expressed as log10 of plague forming units per vial. The experimental results presented by Allison at al. [52] obtained at 31, 37, and 45 °C were simulated by both, one-, and two steps models [2].

## 4. Conclusions

The prediction of the shelf life of nitrocellulose-based propellants and a model vaccine was performed by an advanced kinetic analysis based on discontinuously collected experimental points. It was achieved by modifications of the often applied kinetic and model selection approaches for the phenomenological description of the reaction course versus time. A specific model selection tool was required because the available data were in the form of sparse experimental points. The criteria for comparing models were based on Akaike and Bayesian information theory. The presented results confirmed that the applied procedure helped in balancing between the goodness of fit, the number of parameters, and the models to be used. The results of the simulation of the shelf lives of the considered materials clearly indicate that the application of simplified kinetic models (it is often assumed that the investigated reaction course can be sufficiently well described by 0th or 1st order models) do not result in the correct prediction of their long-term stability. Even worse, depending on the applied test method, these simplified models result in an underestimation (Figure 1) or overestimation (Figure 2 and Figure 4 for the propellant and Figure 7 for the vaccine) of the shelf life. The correct kinetic evaluation of a material’s behavior is therefore of great importance from both safety and financial points of view. Our results show that the prediction of shelf life can be based on the results collected by any analytical technique: For the propellant, the simulations were conducted using data from pressure firing, gas evolution, stabilizer concentration, and heat flow calorimetry techniques. In the kinetic analysis of the vaccine, determination of the rate of the loss of virus infectivity (infectious titer) was used.

We implemented the kinetic parameter evaluated by the advanced kinetic approach into a programmable data logger, i.e., an electronic device that records chosen specific parameters (such as temperature or humidity) over time. Collection of the temperature–time dependences with a chosen frequency (once an hour, day or week) introduced into the kinetic simulation procedure led to continuous estimation of the aging degree. Due to the fact that such a procedure continuously supplies the current degree of deterioration, it has major advantages compared with the common case, in which data loggers record only the temperature–time dependence.

Knowledge of the real rate (not determined by simplified kinetics) of the aging process of any goods allows the prediction of the time–reaction extent dependence for any arbitrarily chosen temperature fluctuation. More generally, the presented procedure enables the estimation of the long-term behavior of any material whose property changes may be expressed by both the kinetic analysis and temperature–time dependences provided by data loggers. The described approach is universal and can be applied for any goods, any methods of shelf life determination, and any type of data loggers.

## Figures and Tables

**Table 1:** The statistical AIC and BIC weights, sum of residual squares RSS, number of data and parameters used in simulations, initial and final values of measured quantities (*Y_init_* and *Y_end_*), and the evaluated kinetic parameters (activation energy, *E*; pre-exponential factor, *A*; reaction order exponents, *n* and *m*) calculated for the testing methods, PF, VST, UPLC, and HFC, respectively. For each method, the statistic and kinetic parameters were calculated for the fixed integer reaction order exponents, *n* = 0, 1, 2, and 3, and for adjustable fitted *n* and *m* values. The results for the best models according to AIC and BIC criteria are displayed in bold. Only in one case was the reaction order model with a fixed integer *n* value (PF, *n* = 3) the best from the statistical point of view. Interpretation of the AIC and BIC criteria are explained in detail in [2].

	wAIC (%)	wBIC (%)	No. of param.	No. of data	RSS	E (kJ·mol^−1^)	Ln(A*s) (-)	n (-)	m (-)	Y_init_	Y_end_
PF	**78.59**	**82.22**	**2**	**15**	**6.66 × 10^4^**	**206.5**	**58.30**	**3**	**0**	**3599**	**5000**
	12.31	8.25	3	15	6.61 × 10^4^	202.8	56.95	2.85	0	3599	5000
	9.09	9.51	2	15	8.88 × 10^4^	179.5	48.52	2	0	3599	5000
	~0	~0	2	15	2.15 × 10^5^	147.4	36.96	1	0	3599	5000
	~0	~0	2	15	9.81 × 10^13^	129.0	30.19	0	0	3599	5000
VST	**59.1**	**56.46**	**3**	**14**	**1.37 × 10^−1^**	**143.1**	**35.30**	**0**	**0.20**	**0.34**	**5**
	25.67	24.53	3	14	1.54 × 10^−1^	141.6	34.10	–1.33	0	0.34	5
	9.66	12.42	2	14	2.36 × 10^−1^	146.9	36.16	0	0	0.34	5
	5.15	6.06	4	14	1.35 × 10^−1^	144.0	35.88	0.57	0.27	0.34	5
	~0	~0	2	14	3.75 × 10^−1^	150.9	37.72	1	0	0.34	5
	~0	~0	2	14	5.47 × 10^−1^	154.7	39.18	2	0	0.34	5
	~0	~0	2	14	7.33 × 10^−1^	158.4	40.61	3	0	0.34	5
UPLC	**55.51**	**48.10**	**3**	**14**	**9.62 × 10^−3^**	**145.8**	**37.13**	**0.62**	**0**	**0.61**	**0**
	44.47	51.88	2	14	1.32 × 10^−2^	148.2	38.16	1	0	0.61	0
	~0	~0	2	14	4.05 × 10^−2^	157.6	41.89	2	0	0.61	0
	~0	~0	2	14	7.37 × 10^−2^	169.1	46.31	3	0	0.61	0
	~0	~0	2	14	6.71 × 10^−1^	150.0	38.27	0	0	0.61	0
HFC	**99.59**	**98.68**	**4**	**28**	**6.71 × 10^2^**	**138.6**	**30.50**	**−4.79**	**0.25**	**0**	**4000**
	0.40	1.31	3	28	1.10 × 10^3^	137.6	30.80	0	0.37	0	4000
	~0	~0	3	28	1.83 × 10^3^	143.3	30.84	–12.40	0	0	4000
	~0	~0	2	28	1.92 × 10^4^	164.8	38.43	0	0	0	4000
	~0	~0	2	28	2.13 × 10^4^	166.5	38.03	1	0	0	4000
	~0	~0	2	28	2.37 × 10^4^	168.2	39.63	2	0	0	4000
	~0	~0	2	28	2.58 × 10^4^	169.9	40.23	3	0	0	4000
